# Molecular Epidemiology of Chronic *Pseudomonas aeruginosa* Airway Infections in Cystic Fibrosis

**DOI:** 10.1371/journal.pone.0050731

**Published:** 2012-11-28

**Authors:** Nina Cramer, Lutz Wiehlmann, Oana Ciofu, Stephanie Tamm, Niels Høiby, Burkhard Tümmler

**Affiliations:** 1 Clinical Research Group, Clinic for Pediatric Pneumology, Allergology and Neonatology, Hanover Medical School, Hanover, Germany; 2 Department of International Health, Immunology and Microbiology, Faculty of Health Sciences, and Department of Clinical Microbiology, University Hospital, Rigshospitalet, University of Copenhagen, Copenhagen, Denmark; 3 Biomedical Research in Endstage and Obstructive Lung Disease Hannover (BREATH), Member of the German Center for Lung Research, Hanover, Germany; University of Duisburg-Essen, Germany

## Abstract

**Background/Methods:**

The molecular epidemiology of the chronic airway infections with *Pseudomonas aeruginosa* in individuals with cystic fibrosis (CF) was investigated by cross-sectional analysis of bacterial isolates from 51 CF centers and by longitudinal analysis of serial isolates which had been collected at the CF centers Hanover and Copenhagen since the onset of airway colonization over 30 years.

**Results:**

Genotyping revealed that the *P. aeruginosa* population in CF is dominated by a few ubiquitous clones. The five most common clones retrieved from the CF host also belonged to the twenty most frequent clones in the environment and in other human disease habitats. Turnover of clones in CF airways was rare. At the Hanover clinic more than half of the patient cohort was still harbouring the initially acquired clone after twenty years of airway colonization. At the Copenhagen clinic, however, two rare clones replaced the initially acquired individual clones in all but one patient.

**Conclusion:**

The divergent epidemiology at the two sites is explained by their differential management of hygiene and antipseudomonal chemotherapy. Hygienic measures to prohibit patient-to-patient transmission and the modalities of antipseudomonal chemotherapy modify the epidemiology of the chronic *P. aeruginosa* infections in CF.

## Introduction

Cystic fibrosis (CF) is a life-limiting monogenic disorder of ion transport of exocrine glands that is caused by mutations in the CF transmembrane conductance regulator (*CFTR*) gene [1]. The basic defect predisposes to chronic bacterial airway infections, particularly with *Pseudomonas aeruginosa* [1], [2]. The CF host is immunocompromised by impaired airway clearance [3], [4]. Early antipseudomonal chemotherapy is often successful to clear the airways from *P. aeruginosa* [5], [6], however, once *P. aeruginosa* has taken chronic residence in the CF lungs, the bacterium becomes resistant to eradication by chemotherapy [7].

During chronic infection *P. aeruginosa* develops common features in CF airways [8]. The bacteria diversify in morphotype, often become immotile, modify their LPS structure and reduce the production of siderophores and virulence effectors [8]. Late isolates, however, do not differ from early and intermediate clonal isolates in their capacity to establish chronic infection and cause extensive inflammation in the respiratory tract [9].

The course of the chronic *P. aeruginosa* infections in CF has been followed by pheno- and genotyping of serial isolates [10]–[14]. Most studies focused either on a few consecutive years of the infection [10], [12]–[14] or on the comparison of some early, intermittent and late isolates [9], [15]. The authors have collected serial *P. aeruginosa* isolates from CF patients since the onset of colonization in the 1970s or 1980s [11], [16]. Genotyping of these strain collections from the CF clinics Copenhagen and Hanover provided the unique opportunity to compare the molecular epidemiology of a chronic bacterial infection in a susceptible human host at two sites over a 30-year time period. The spectrum of patients‘ *P. aeruginosa* clones identified at the two CF clinics was compared with that of isolates from the environment, other infections with *P. aeruginosa* and CF patients seen at other clinics. The combined approach provided insight into the global population biology of *P. aeruginosa* in CF and the (site-specific) course of the chronic infection in the individual patient.

## Materials and Methods

### Ethics Statement

The study has been approved by the Ethics Committee of Hanover Medical School (study no. 3739). Written informed consent was obtained from patients and their parents.

**Figure 1:**
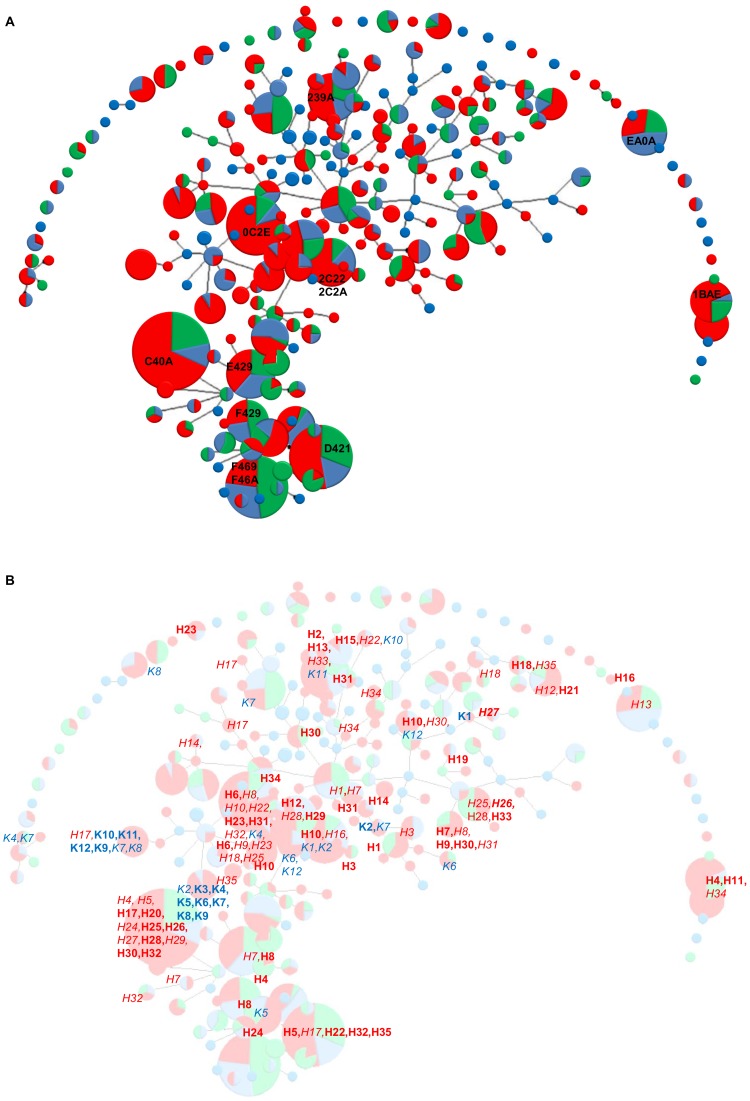
Distribution of CF airway isolates in the *P. aeruginosa* population. A. Clonal complex structures of 955 *P. aeruginosa* strains collected from individuals with cystic fibrosis (red), other human infections (green) and from the inanimate environment (blue). The diameter of the circle is proportional to the number of isolates with this genotype. Clonal complexes were calculated from the 15-marker genotype of the core genome by the eBurst algorithm. The ten most frequent clones are marked by their hexadecimal code. ExoU- and exoS-positive clones that share the same 15-marker genotype such as F469 and F46A or 2C22 and 2C2A are lumped together. B. Localization of the genotypes of the serial isolates collected from individuals with CF from the Hanover (H1-H35) and Copenhagen clinics (K1-K12) mapped onto the overall population structure (Fig. 1A) shown in the background. Persistent clones are marked in bold font, sporadic clones are shown in italics.

### Strain Collection

The *P. aeruginosa* strain collection examined consisted of the following: Isolates from individuals with CF were collected at the CF centers in Aachen, Athens, Belfast, Bremen, Brussels, Cologne, Copenhagen, Cork, Den Haag, Dublin, Düsseldorf, Erlangen, Frankfurt, Geneva, Ghent, Gießen, Graz, Halle, Hamburg, Hanover, Heidelberg, Hobart, Homburg, Innsbruck, Jena, Leuven, Liverpool, Linz, London, Lund, Lyon, Karlsruhe, Madrid, Magdeburg, Milan, Montpellier, Münster, Oldenburg, Paris, Poznan, Rostock, Rotterdam, St. Gallen, Sydney, Utrecht, Torino, Tübingen, Verona, Vienna, Zurich and Zwickau. The COPD isolates were collected from patients who were seen at the Buffalo Veteran Medical Center, N.Y [17]. The ICU isolates were collected from intubated patients with ventilator-associated pneumonia at 13 hospitals in Europe [18]. Isolates from patients with corneal ulcers were collected from six hospitals in England [19]. The environmental isolates were collected from sanitary facilities in households in Germany, the rivers Aller, Oker, Rhein, Ruhr, and the deep sea south of Japan [20], [21]. In addition strains from the ATTC and DSMZ collections of unknown origin were included in the study.

**Figure 2:**
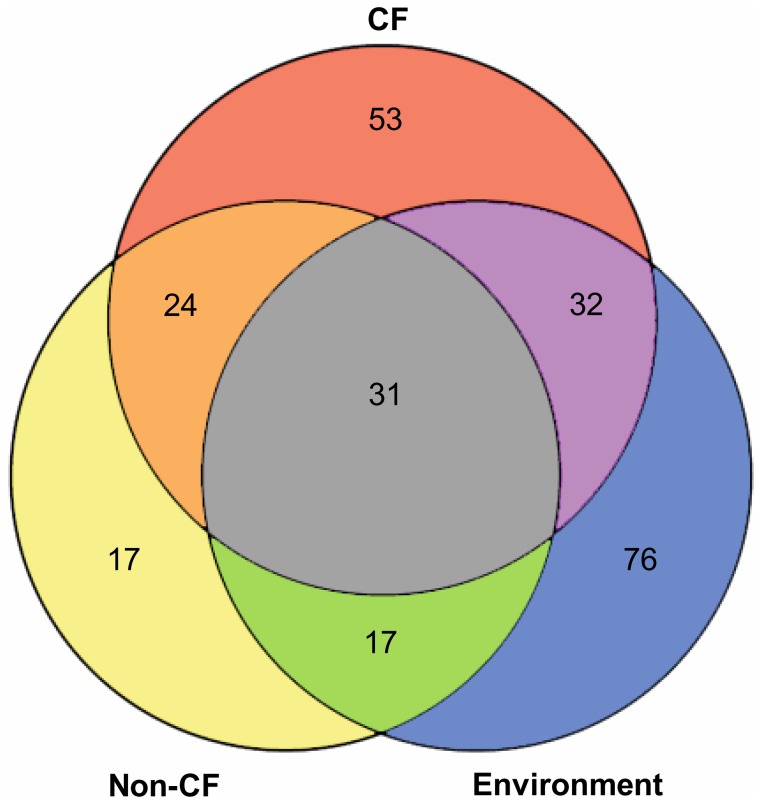
Venn diagram of the number of *P. aeruginosa* genotypes retrieved from patients with CF, other human infections and the inanimate environment. The intersections indicate the number of shared clones.

Serial isolates. *P. aeruginosa* isolates had been recovered from respiratory secretions (bronchoalveolar lavage, sputum, deep throat swabs) of individuals with CF who had been regularly seen at the CF clinics in Copenhagen or Hanover since the age of diagnosis and who became positive for *P. aeruginosa* prior to 1990. *P. aeruginosa* had been identified by conventional biochemical tests. Secondary subcultures were stored as glycerol stock cultures at –80°C. At both sites isolates had been stored since the onset of airways’ colonization of the individual patients with *P. aeruginosa*. At the Hannover site isolates were regularly collected in half year intervals, whereas at the Copenhagen site such a regular timeline did not exist.

**Figure 3:**
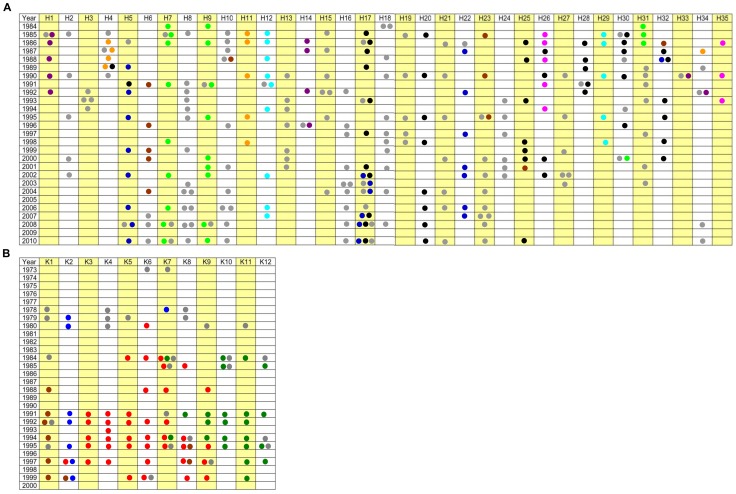
Longitudinal follow-up of *P. aeruginosa* genotypes in CF lungs. Course of chronic airway infections with *P. aeruginosa* since onset of colonization in patients with CF seen at the CF clinics Hannover (H1– H35, Fig. 3A) and Copenhagen (K1– K12, Fig. 3B). The most common clones are marked in different colours (eight for Hannover and four for Copenhagen), all other clones are marked in grey (Hanover: black =  clone C40A, light green =  clone 7C9A, light blue =  clone 3C2A, orange =  clone 1BAE, pink =  clone 6D92, blue =  clone D421, brown =  clone 0C2E, purple =  clone AC9A; Copenhagen: red =  clone 6822, green =  clone 4022, brown =  clone 2C22, blue =  clone AE9A). If no isolates were available from the Hannover CF clinic in 2010, patients had previously moved to another region (H2, H22, H23, H24, H27, H30, H31, H32), had passed away or had received a lung transplantation (H1, H3, H4, H11, H13, H14, H19, H26, H28, H29). Patients H33, H34 and H35 were only transiently colonized with *P. aeruginosa.* At the Copenhagen clinic 5 patients got a lung transplantation between 1996 and 2004, 5 other patients died (1999–2008). One of the deceased patients had also a lung transplantation, so that in total 9 patients have either died or received a lung transplantation.

### *P. aeruginosa* Genotyping

*P. aeruginosa* strains were genotyped by a custom-made microarray following the published previously protocol [22]. The relatedness of strains by multilocus genotype was calculated by the eBurst algorithm (http://eburst.mlst.net) [23]–[25].

**Figure 4:**
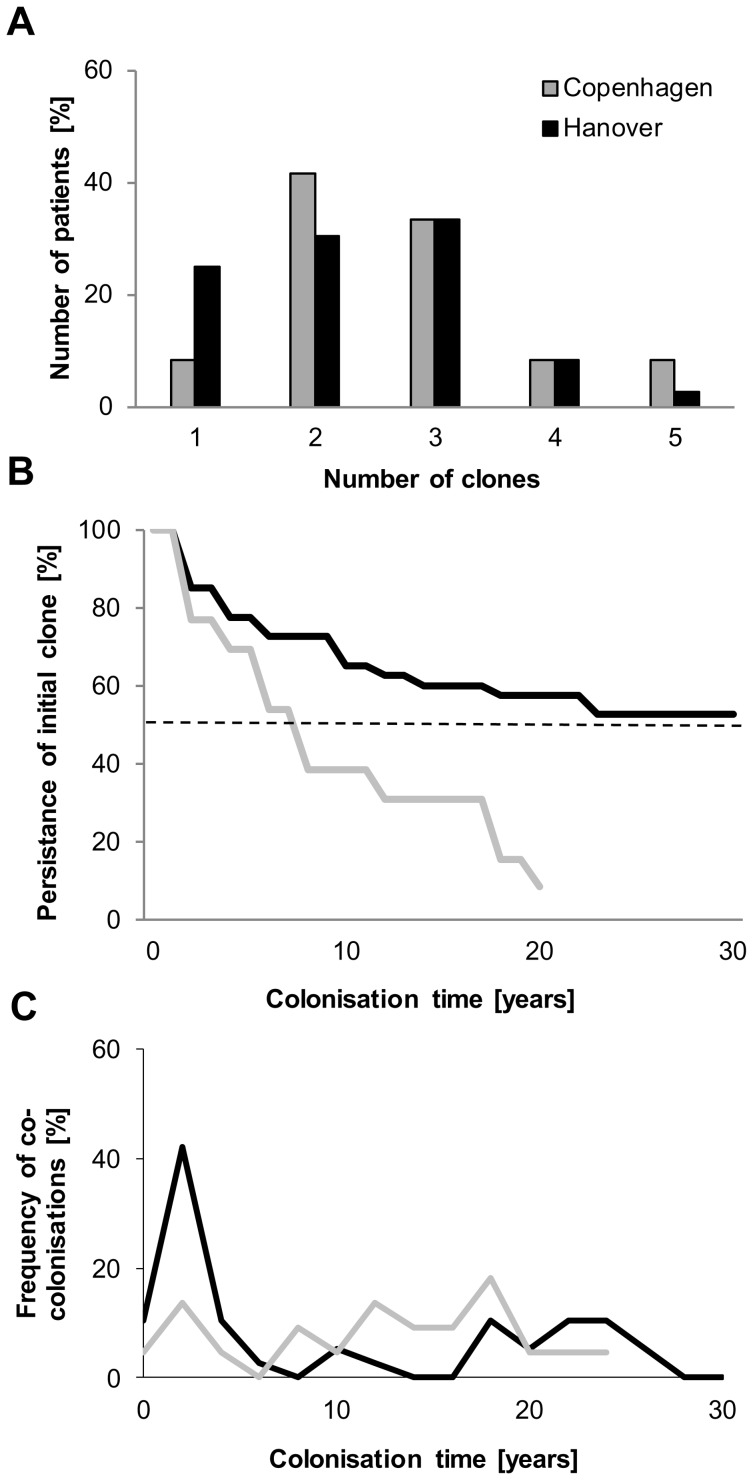
Diverging epidemiology of the *P. aeruginosa* infection at two CF centers. Cumulative comparison of the course of the infection with *P. aeruginosa* in the patient cohorts from the CF clinics Hannover and Copenhagen. A. Number of genotypes detected in the individual patients during the observation period. B. Persistance of the initially acquired clone. C. Frequency of co-colonizations with two or more clones during the observation period.

### Clinical Data

Information on patient characteristics (gender, date of birth, *CFTR* mutation genotype; age at onset of colonization with *P. aeruginosa*; Supporting Information, Table S3) and on antipseudomonal chemotherapy was extracted from the local CF patient databases and the patients‘ records. The two cohorts of CF patients from Copenhagen and Hanover were followed for the same observation period of 27 years.

**Figure 5:**
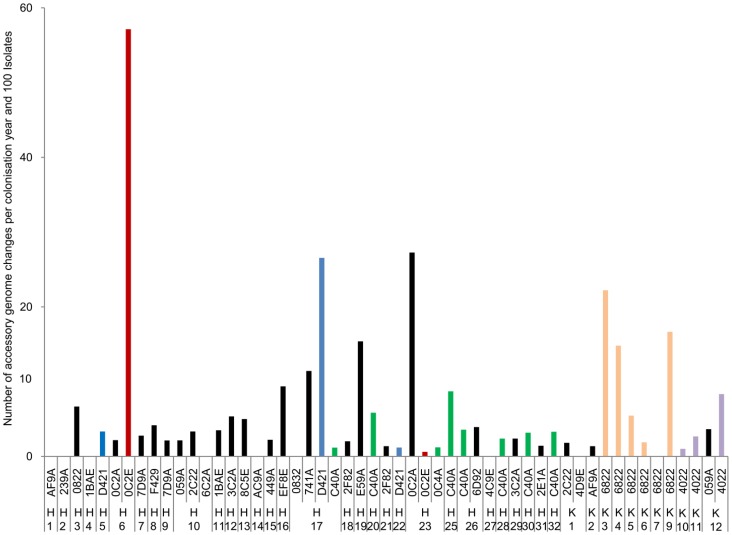
Microevolution of the accessory genome. The bars indicate the number of changes of markers of the accessory genome for each persistant clone (5 isolates or more) in patients H1– H23, H25– H32, K1– K7 and K9- K12. The number of changes was normalized by year and 100 isolates.

Patients received aerosol, oral and/or intravenous antipseudomonal chemotherapy, but they were not subject to eradication therapy of the first detection of *P. aeruginosa* [7] which was established at both clinics at a later point in time.

**Figure 6:**
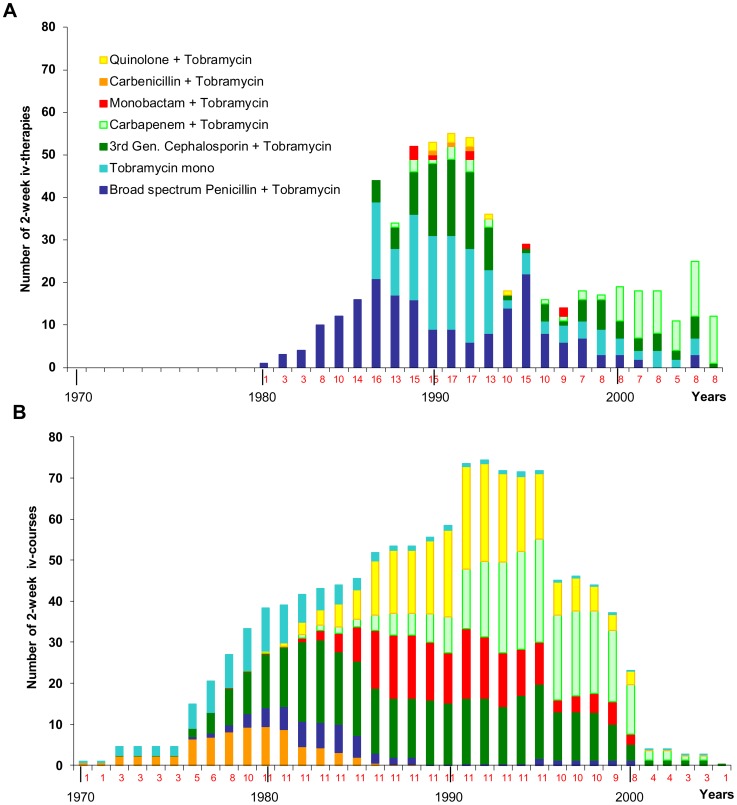
Antipseudomonal chemotherapy at the two CF clinics Hanover and Copenhagen. Number of the annual courses of intravenous antimicrobial chemotherapy administered to the 35 and 12 CF patients from the Hannover and Copenhagen clinics, respectively. The number of treated patients is indicated for each year below the abscissa. The various classes of antimicrobials are differentiated by colour.

## Results

### Population Biology of *P. aeruginosa* in the CF Lung Habitat

A collection of 955 *P. aeruginosa* isolates of independent origin was genotyped with a low-resolution microarray that represents the conserved core genome with 16 informative SNPs and the variable accessory genome with 42 marker genes [22], [26]. 454 strains had been isolated from 295 CF patients from 51 CF centers in Europe or Australia. The other 501 strains were collected from inanimate aquatic environments or from patients suffering from ventilator-associated pneumonia (VAP), corneal ulcers or chronic obstructive pulmonary disease (COPD), respectively (Supporting Information, Table S1).

Microarray typing revealed that the 955 independent strains belonged to 248 genotypes (Figure 1). The ten most frequent clones made up 31.1% of the strain collection. In contrast, 126 clones were represented by only one isolate and 37 clones by two isolates. This finding validates our earlier observation on a smaller set of strains [22] that the *P. aeruginosa* population consists of many rare clones and a few globally distributed major clones.

The e-Burst algorithm [23], [24] grouped the genotypes into 41 singletons, five small clonal complexes and one very large group (Figure 1). 187 genotypes, i.e. 75.4% of total, were linked into this highly branched eBURST group. Such a scenario of singletons and of one large group is typical for populations with high recombination to mutation ratios [24].

The ten most common clones in CF lungs are designated in Figure 1 by hexadecimal code. The distribution of the clones recovered from CF patients overlapped with those from the environment and the other disease habitats. Consistent with a previous report [27] there was no evidence for a widespread CF transmissible clone. The five most common *P. aeruginosa* clones in the CF population also belonged to the ten most common clones in the environment and in VAP, COPD and keratitis (Fig. 1A, Table S1). The ubiquitous ExoS-positive clone C [28] and the ExoU-positive clone PA14 [22], [29] were the most abundant clones in CF airways.

The Venn diagram in Figure 2 summarizes the distribution of the clonal complexes in the three habitats. Thirty-one clones were retrieved from all three habitats. These 31 clones represented 12% of all clones and 79% of all isolates demonstrating that these clones are abundant and ubiquitous. Fifty-eight percent of clones were only identified in one habitat, but all but two of these 146 clones were infrequent or rare. In summary, the very same few clones were dominant in the *P. aeruginosa* population in all investigated habitats (Fig. 1).

Comparison of the epidemiology of *P. aeruginosa* infections at two CF centers by typing serial isolates since onset of colonization in the 1970s to 1980s.

The global analysis of single CF isolates per patient from many CF centers revealed a distribution of *P. aeruginosa* clones that is similar to that found in VAP, COPD and aquatic inanimate habitats. We wanted to know how this global view translates into the individual patient’s *P. aeruginosa* infection course in terms of persistence, co-colonization and turnover of clones. The CF clinics in Copenhagen (Denmark) and Hanover (Germany) maintain unique strain collections of serial isolates from individual patients taken at regular intervals since the first detection of *P. aeruginosa* going back to the 1970s (Copenhagen) or 1980s (Hanover).

Figure 1B shows the clonal distribution of the 789 serial *P. aeruginosa* isolates that were available from 47 patients who had been regularly seen since age at diagnosis at the Hanover (35 patients) or Copenhagen clinics (12 patients). The frequent clones in the Hanover populations that were isolated from two or more patients belonged also to the most common clones in the global *P. aeruginosa* population (Fig. 1B, Fig. 3). The Copenhagen strain population, however, was dominated by the two related clones 6822 and 4022. These clones are rare in the global population (Fig. 1B, Fig. 3).

Strains have been continuously collected at the Hanover site during the whole study period from 1984–2010. If the last genotyped isolate had been taken before 2010, the patients had either died (n = 11), had received a lung transplant (n = 2) or had left the clinic because of change of residence (n = 6). At the Copenhagen site one of the 12 patients had died and another six patients underwent lung transplants.

Of the 35 patients from the Hanover clinic, three patients spontaneously lost their initially acquired clone after six to 75 months of chronic colonization, of whom one became re-colonized with another clone 17 years later (Fig. 3, Supporting Information, Table S2). The other 32 patients became chronically colonized with *P. aeruginosa* and maintained their initially acquired clone for a prolonged period of time (see below). In contrast, at the Copenhagen clinic two major clones emerged in individual patients in 1980 (6822, red or DK-1) and 1984 (4022, green or DK-2) [30] and were then transmitted over the years to all but one patient (Fig. 3, Table S2). In summary, the sharing of clones was significantly more frequent in the Copenhagen than in the Hannover cohort (*P* = 0.011, Fisher’s exact test). Superinfection was not detectable in the Hanover cohort, but common in the Copenhagen group as reported previously [31], [32], [33].

During the observation period patients harboured one to five *P. aeruginosa* clones (Fig. 4A). Two or three unrelated genotypes were detected in the majority of patients. Only one clone was identified in a quarter of the Hanover group, whereas just two of the 12 patients from the Copenhagen clinic were continuously colonized with just one clone.

The discordant epidemiology is also demonstrated by the persistence of the initially acquired *P. aeruginosa* clone (Fig. 4B). More than half of the Hanover cohort carried the same clone during the whole observation period of 30 years since the onset of colonization. At the Copenhagen clinic the median carriage of the initial clone was eight years. By the 20^th^ year of the *P. aeruginosa* infection all patients but one of the Copenhagen cohort had lost their first clone (Fig. 4B). The pattern of co-colonization with more than one clone was also different at the two sites (Fig. 4C). At the Hanover clinic co-colonization peaked during the second and third years of colonization, and at the Copenhagen clinic the annual frequency of co-colonisation fluctuated between 0 and 20%. Thus in the Hanover group the competition of bacterial clones to take residence in the CF airways took place during the initial period of the chronic infection, whereas a continuously ongoing competition occurred in the Copenhagen patients‘ airways. The initially acquired genotypes were replaced by two major clones (DK-1 and DK-2) [34] that are rare in the global *P. aeruginosa* population (compare Figures 1A and 1B).

### Microevolution of the Accessory *P. aeruginosa* Genome during CF Lung Infection

So far the structure of the *P. aeruginosa* populations infection of CF patients‘ lungs was described in terms of turnover and persistence of clones, but not in terms of the intraclonal microevolution of the accessory genome [26]. The microarray can detect the mobility of the accessory genome of the serial isolates (Table S2) by the loss or acquisition of markers that are representative for the common genomic islets islands of the *P. aeruginosa* pangenome [22], [35]. Figure 5 compares the dynamics of the accessory genome of chronically carried clones in the CF lungs. The flexibility of the individual clones differed dramatically from each other. Nine clones remained invariant in their marker composition during chronic colonization of the patient’s airways. In contrast eight clones strongly diversified in their accessory genome over time. Of the common clones found in the Hanover (OC2E, D421, C40A) and Copenhagen CF populations (6822, 4022), only clone C exhibited a similar rate of microevolution in the seven carriers. The other four *P. aeruginosa* clones, however, showed large variations of the mobility of their accessory genome in the individual CF hosts (Fig. 5). OC2E, the third most common clone in the global population, represented the extreme. The highest rate of bacterial microevolution was detected in CF host H6, but virtually no changes occurred in patient‘s H22 lungs. This data suggests that the individual CF habitat (host phenotype) plays an important role in intraclonal genome evolution of *P. aeruginosa.*

## Discussion

Airway infections by *P. aeruginosa* in individuals with CF are a paradigm for chronic bacterial infections in a susceptible human host. Here we report on the retrospective analysis of unique serial isolate strain collections that were taken at two clinics from CF patients since the onset of airway colonization over a 30-year period. To differentiate between site-specific and common features of the evolution of the *P. aeruginosa* populations in the two cohorts, a cross-sectional analysis was performed on CF isolates from 51 CF centers that had been collected between 1980 and 2010. Moreover, we compared the population biology of *P. aeruginosa* in CF airways with that in the inanimate environment and in other human infectious diseases.

Genotyping revealed that the *P. aeruginosa* population is dominated by a few ubiquitous clones. The five most common clones retrieved from the CF host also belonged to the twenty most frequent clones in the environment and in other human disease habitats. If clones had only been detected in CF patients, their frequency was low or very low. However, the comparative typing analysis of the serial isolates from the CF centers in Hanover and Copenhagen taught us that site-specific expansion of clones does exist. At the Hanover site the clonal distribution was indistinguishable from that of the global distribution in CF airways. At the Copenhagen clinic, however, two clones had replaced the initially acquired individual clones in all but one patient. These two successful clones belong to the same clade and were rare in other habitats (see Fig. 1). Thus two uncommon clones were selected in the Copenhagen CF population. This scenario did not occur in the Hanover CF population. At the Hanover site more than half of the cohort maintained their initially acquired clone throughout the whole observation period. Turnover and replacement of clones was rare and occurred predominantly during the first two years of colonization.

The different management of the *P. aeruginosa* infections at the two CF clinics in terms of antimicrobial chemotherapy and infection control is probably responsible for the discordant epidemiology in the two cohorts. The Copenhagen clinic provided more courses of 2-week iv antipseudomonal chemotherapy than the Hannover CF clinic (Figure 6). The choice of antipseudomonal agents shifted over the years from initially carbenicillin to broad-spectrum penicillins, third-generation cephalosporins, monobactams and finally carbapenems and fluoroquinolones. This wave-like prevalence of antimicrobials was far less pronounced in the regimen of the Hanover clinic. Antipseudomonal iv chemotherapy was less frequent and the prescription of drugs more conservative at the Hanover clinic (Figure 6). Tobramycin alone or in combination with a broad-spectrum penicillin or a third-generation cephalosporin was the standard treatment for close to 20 years. Only by 2000 carbapenems became the dominant antipseudomonal agents for the meanwhile adult patient population.

The comparison of the prescription attitudes at the two clinics suggests that the high antimicrobial load and the continuous change of the preferred antimicrobial at the Copenhagen clinic exerted strong selection pressures on the *P. aeruginosa* populations in the CF airways which probably facilitated the replacement of the initially acquired clones by two major clones. These clones were apparently equipped with superior fitness traits to cope with antimicrobial pressure.

Since the two major Copenhagen clones are rare in the global *P. aeruginosa* population, they probably spread by nosocomial superinfection. The Copenhagen CF clinic (like the Hannover CF clinic) separated *P. aeruginosa* – positive and *P. aeruginosa* – negative patients from each other, but during iv antipseudomonal treatment the *P. aeruginosa* - positive patients were accommodated in a day hospital – like ward where the patients could communicate with each other. Such a scenario allowed for patient-to-patient transmission. In contrast, no superinfection was observed at the Hannover site where stringent infection control did not only apply to the separation of *P. aeruginosa* – positive from *P. aeruginosa* – negative patients, but also to the inpatient antipseudomonal chemotherapy. A *P. aeruginosa* – positive patient was accommodated in a one-bed cubicle to receive iv antimicrobial therapy and was kept separate from other *P. aeruginosa* - positive CF patients at the ward for infectious disease. Although our study due to its retrospective nature cannot provide definitive proof, the data strongly suggest that the hygienic precautions to prohibit patient-to-patient transmission and the modalities of antipseudomonal chemotherapy modify the epidemiology of the chronic *P. aeruginosa* infections in CF.

The results of our study have not only hands-on implications for CF infectiology, but also provide insight into the population structure of *P. aeruginosa*. The eBurst program generated one large convoluted group that incorporated most *P. aeruginosa* clones. This type of population snapshot has only been observed in bacterial taxa with high recombination to mutation ratios [24]. The ancestor-descendant links in this large eBURST group have to be taken with caution, but the eBurst diagram nevertheless provides strong evidence that the evolution of the genome in the *P. aeruginosa* population is more driven by recombination than by mutation. The *P. aeruginosa* genome is made up of a conserved core genome with just 0.5% interclonal sequence diversity and a flexible accessory genome. These ‚regions of genome plasticity‘ (RGP) [26] consist of genome islets and genomic islands [35]. Linkage disequilibrium between core genome and RGPs is typical for a *P. aeruginosa* clone, but nevertheless some uptake and loss of RGPs causes intraclonal genome diversity [22], [35]. Our genotyping of RGP markers unravelled a large gradient of accessory genome mobility in the serial isolates from CF patients‘ lungs. A few clones remained almost invariant in their RGP marker profile during chronic colonization of the CF habitat, whereas others exhibited strong fluctuations. The same clone behaved differently in different hosts, but also co-existing clones behaved differently in the same CF host (Fig. 5). CF lungs are continuously remodelled by infection, inflammation, remodeling and fibrosis [36]. The intraluminal bacteria are exposed to deep gradients of oxygen and nutrients [37] and there is a continuously on-going host-pathogen interaction. Thus, CF lungs are a spatiotemporally highly heterogeneous habitat which corresponds to the observed strong clone-to-clone and host-to-host variation of the microevolution of the accessory genome. Thus many evolutionary trajectories of how the bacteria adapt to the CF lung habitat seem to exist. Sequencing of serial isolates – as it has already been performed for a few selected cases [16], [34], [38] – may unravel the main targets and the major modes of the microevolution of *P. aeruginosa* to infiltrate and persist in CF lungs.

## Supporting Information

Table S1 **Origin and genotype of the 955** ***P. aeruginosa*** **strains subjected to eBURST Analysis.**
(XLS)

Table S2 **Longitudinal course of** ***P. aeruginosa*** **genotypes of patients' respiratory isolates from the CF clinics Hanover and Copenhagen.**
(XLS)

Table S3 **Characteristics of study participants.**
(XLS)
